# Edge Intelligence-Based Rail Transit Equipment Inspection System

**DOI:** 10.3390/s26010236

**Published:** 2025-12-30

**Authors:** Lijia Tian, Hongli Zhao, Li Zhu, Hailin Jiang, Xinjun Gao

**Affiliations:** 1 State Key Laboratory of Advanced Rail Autonomous Operation, Beijing Jiaotong University, Beijing 100044, China; 24125163@bjtu.edu.cn (L.T.); lizhu@bjtu.edu.cn (L.Z.); lhjiang@bjtu.edu.cn (H.J.); 2 Signal and Communication Research Institute, China Academy of Railway Sciences Group Co., Ltd., Beijing 100081, China; tkygxj@163.com

**Keywords:** edge intelligence, rail transit, intelligent inspection, computer vision, 5G, object detection, YOLOv8

## Abstract

The safe operation of rail transit systems relies heavily on the efficient and reliable maintenance of their equipment, as any malfunction or abnormal operation may pose serious risks to transportation safety. Traditional manual inspection methods are often characterized by high costs, low efficiency, and susceptibility to human error. To address these limitations, this paper presents a rail transit equipment inspection system based on Edge Intelligence (EI) and 5G technology. The proposed system adopts a cloud–edge–end collaborative architecture that integrates Computer Vision (CV) techniques to automate inspection tasks; specifically, a fine-tuned YOLOv8 model is employed for object detection of personnel and equipment, while a ResNet-18 network is utilized for equipment status classification. By implementing an ETSI MEC-compliant framework on edge servers (NVIDIA Jetson AGX Orin), the system enhances data processing efficiency and network performance, while further strengthening security through the use of a 5G private network that isolates critical infrastructure data from the public internet, and improving robustness via distributed edge nodes that eliminate single points of failure. The proposed solution has been deployed and evaluated in real-world scenarios on Beijing Metro Line 6. Experimental results demonstrate that the YOLOv8 model achieves a mean Average Precision (mAP@0.5) of 92.7% ± 0.4% for equipment detection, and the ResNet-18 classifier attains 95.8% ± 0.3% accuracy in distinguishing normal and abnormal statuses. Compared with a cloud-centric architecture, the EI-based system reduces the average end-to-end latency for anomaly detection tasks by 45% (28.5 ms vs. 52.1 ms) and significantly lowers daily bandwidth consumption by approximately 98.1% (from 40.0 GB to 0.76 GB) through an event-triggered evidence upload strategy involving images and short video clips, highlighting its superior real-time performance, security, robustness, and bandwidth efficiency.

## 1. Introduction

Rail transit has become one of the most widely adopted modes of transportation worldwide due to its high reliability, large capacity, and punctuality. With the continuous expansion of rail transit networks, ensuring operational safety has become a paramount concern. The normal operation of various rail transit devices is critical to system safety, as any malfunction or abnormal behavior may directly affect service stability and potentially lead to serious incidents.

Traditional inspection methods in rail transit systems rely heavily on manual labor. This “person-to-equipment” management approach has revealed numerous limitations as network scales increase. Manual inspections are inherently labor-intensive, time-consuming, and costly. For example, some studies [1] estimate that manual inspection teams may incur annual costs of several million dollars, while visual inspection tasks exhibit human error rates as high as 2.23%. Furthermore, inspection accuracy can degrade under fatigue or in complex environments, leading to omissions and misidentifications. These issues ultimately compromise reliability, efficiency, and timeliness, posing potential safety hazards to rail transit operations.

To improve inspection efficiency and safety, researchers have proposed a variety of smart inspection solutions. Yue et al. developed a QR code-based inspection system [2,3], which simplifies data recording but still requires on-site operation. Chen et al. designed an autonomous inspection robot for metro communication and signal rooms to achieve real-time monitoring and control. Wang et al. adopted rail-based inspection robots for automatic inspections, reducing labor costs, but the high investment in equipment limits their wide application [4,5]. Hu et al. applied AI-based video recognition technology for automatic inspection of rail transit signal devices, and Wang et al. proposed a wayside system for diagnosing freight wagon performance [6]. However, these video-based solutions often suffer from long processing times, low efficiency, or high false alarm rates, limiting their field application. Huang et al. used the YOLOv5 algorithm for automatic identification of device indicator lights, improving inspection efficiency and reducing missed checks.

Computer vision (CV), widely applied in object detection and image recognition [7,8,9], has the potential to greatly enhance the automation and precision of rail transit equipment inspection [10,11]. However, most rail systems rely on centralized management with limited computing resources, creating latency challenges for CV algorithms that require substantial computational power. Prolonged inference time severely restricts real-time applicability. To mitigate these challenges, edge computing (EC) has been introduced for distributed inference near data sources [12,13]. By processing data on edge nodes instead of centralized servers, EC can reduce network latency and improve response speed to the millisecond level [14].

To further enhance system intelligence and adaptability, this study integrates Edge Intelligence (EI) into the inspection framework [15]. Personnel supervision is considered an integral part of this equipment inspection system, as the validity of manual intervention is a prerequisite for equipment safety. Recent systematic surveys, such as those by Barbuto et al., have highlighted the transformative potential of “disclosing” edge intelligence, moving beyond simple data offloading to distributed, cognitive computing at the edge [16]. Similarly, Liu et al. demonstrated the efficacy of smart traffic monitoring systems utilizing computer vision and edge computing, providing a robust precedent for applying these technologies in transportation verticals [17]. Furthermore, as Ferdowsi et al. emphasize, the deployment of deep learning in intelligent transportation systems requires a rigorous focus on reliability and robustness [18], factors which are central to our system design. While previous studies have theoretically explored the concept of ‘disclosing’ edge intelligence or applied edge computing to general traffic monitoring, these approaches often lack a unified framework tailored for the specific constraints of subterranean rail transit environments. Specifically, existing solutions rarely address the dual challenge of ensuring data privacy via 5G private networks while simultaneously optimizing bandwidth through event-triggered video evidence uploads. Our work bridges this gap by implementing a fully ETSI MEC-compliant architecture that integrates robust deep learning models directly into the inspection workflow, ensuring both real-time anomaly detection and high-efficiency data transmission.

As an emerging paradigm of artificial intelligence (AI), EI enables localized inference, real-time decision-making, and collaborative model deployment at the network edge [19]. Complementing this paradigm, the proposed system adopts an ETSI MEC-compliant architecture that provides standardized ultra-low-latency communication and high-bandwidth data handling, thereby enhancing deployment scalability.

In terms of task execution, we design a CV-based inspection algorithm composed of two modules. The first performs real-time detection of personnel and equipment using object detection [20], while the second analyzes offline inspection videos uploaded by field personnel. Image data are captured by terminal sensors and processed at nearby edge nodes, and aggregated results are transmitted to the central management platform. This distributed approach improves inspection automation and real-time responsiveness while effectively reducing operational costs.

The main contributions of this paper are summarized as follows:

- We propose a rail transit equipment inspection system based on Edge Intelligence and 5G technology, leveraging an ETSI MEC-compliant architecture to overcome the cost and error limitations of manual inspection.
- We develop and quantitatively validate a two-stage inspection algorithm. A fine-tuned YOLOv8 model achieves 92.7% mAP@0.5 for detection, and a ResNet-18 model achieves 95.8% accuracy for status classification, enabling real-time monitoring of rail transit equipment.
- We deploy and evaluate the system in real-world scenarios on Beijing Metro Line 6. Results show that, compared with a traditional cloud-centric architecture, the proposed EI system reduces average end-to-end latency by 45% (from 52.1 ms to 28.5 ms) and reduces uplink bandwidth consumption by 98.1% through an event-triggered mechanism that includes video evidence.

The remainder of this paper is organized as follows. Section 2 introduces the architecture of the proposed EI-based inspection system, including hardware specifications. Section 3 details the real-time monitoring algorithm for personnel and equipment. Section 4 presents experimental results, including latency, model performance, bandwidth efficiency, and field validation. Finally, Section 5 concludes the paper and outlines future work.

## 2. System Model of the Edge Intelligence-Based Equipment Inspection System

In this section, we provide a detailed analysis of the Edge Intelligence-based rail transit equipment inspection system from both the system architecture and operational perspectives.

### 2.1. Equipment Inspection System Architecture

**Figure 1:**
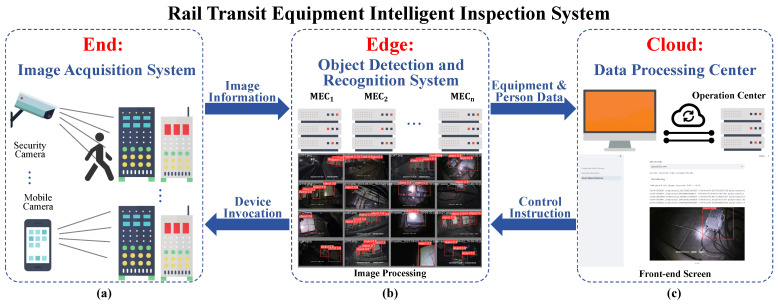
The architecture of the EI-based Rail Transit Equipment Inspection System: (**a**) Terminal Layer; (**b**) Edge Layer; (**c**) Cloud Layer.

The EI-based inspection system is deployed on the EI platform, as shown in Figure 1. The system architecture consists of three functional layers: the terminal layer, the edge layer, and the cloud layer. The computational capabilities of these layers increase from the terminal to the cloud. By using the cloud-edge-end collaborative edge computing system logic [13], we can effectively reduce the amount of data transmitted from the terminal to the cloud (data processing center) [21]. Based on the actual requirements of rail transit equipment inspection tasks and the cloud-edge-end architecture, the system framework consists of the following three main components:

- Terminal Layer (Figure 1a): Cameras are deployed at locations such as trackside, tunnels, and equipment rooms, or carried by inspection personnel to perform tasks related to the collection of equipment and personnel status.
- Edge Layer (Figure 1b): An edge server is installed at each edge node, where the computer vision-based inspection algorithm is deployed. The target detection system on the edge server interacts directly with the image acquisition system at the terminal layer, processing the image data captured and uploaded by cameras, generating status information of the equipment, and uploading it in real-time to the cloud’s data processing center for further analysis.
- Cloud Layer (Figure 1c): A data processing center is deployed in the cloud to aggregate and analyze the data and generate visualizations. The data processing center allows for the real-time monitoring of the work status of inspection personnel and the condition of rail transit equipment, as well as viewing the image data processed by the edge server.

The EI architecture further provides key advantages that significantly enhance the performance and reliability of the equipment inspection system. By processing raw, uncompressed video data directly at the edge, the system preserves fine-grained visual details that would otherwise be lost during cloud transmission, thereby improving detection accuracy. The adoption of a dedicated 5G private network ensures that all inspection data remain isolated from the public Internet, greatly strengthening system security and reducing the risk of data interception or tampering. In addition, the distributed deployment of edge nodes eliminates single points of failure, allowing each node to continue operating independently and ensuring robust system reliability even under partial network or device failures. Finally, by uploading only event-triggered results instead of continuous video streams, the EI system substantially reduces bandwidth consumption and improves communication efficiency, making it highly suitable for long-term, real-time equipment monitoring scenarios.

### 2.2. Operational Workflow of the Intelligent Equipment Inspection System

**Figure 2:**
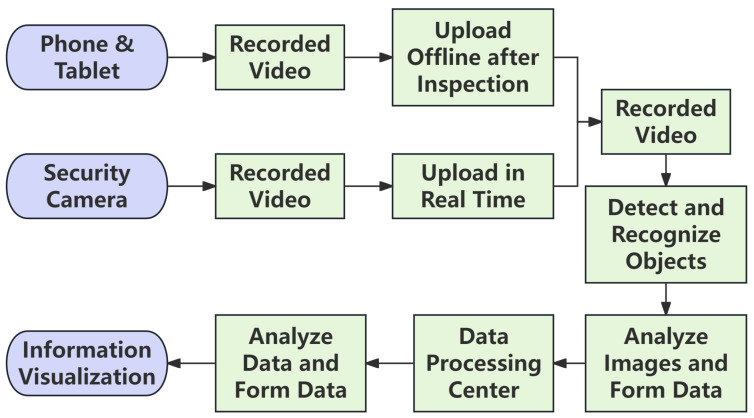
Operational Workflow of the Edge Intelligence-based Equipment Inspection System.

The operational workflow of the edge intelligence-based rail transit equipment inspection system is illustrated in Figure 2. The system workflow starts with the collection of real-time footage or recorded videos by inspection personnel, which is then processed through the edge server and data processing center, enabling efficient, accurate, and reliable equipment inspection. The specific workflow is as follows:

- Terminal Layer: Inspection personnel can record videos using mobile phones or tablets. After completing the inspection, the recorded videos are uploaded to the system. The video upload occurs offline, meaning it is uploaded after the inspection task is completed. Security Camera: Cameras installed at rail transit equipment locations capture real-time footage, which is transmitted to the system. This real-time footage is uploaded immediately to ensure live monitoring.
- Edge Server Layer: The data is transmitted in real-time or offline to the Edge Server, which performs object detection and recognition. Using computer vision techniques, the edge server efficiently analyzes the image data to identify equipment status or potential faults [22]. Our system defines “real-time” as achieving an inference-to-alert latency below 33 ms, enabling a processing speed of 30 FPS. Through image processing and data analysis, the edge server conducts real-time inspection of the equipment, determining whether the equipment is functioning properly or has any anomalies.
- Data Processing Center Layer: The data uploaded by the edge servers is further analyzed at the Data Processing Center in the cloud. The data processing center integrates and analyzes the image data, equipment status, and other relevant information to generate visualizations, enabling real-time monitoring of equipment conditions. The data processing center also further processes the analysis results of equipment status to ensure the efficient execution of the inspection tasks.

This workflow ensures high efficiency in task execution, high precision in equipment recognition, and high reliability in system monitoring, guaranteeing the stable operation of rail transit equipment.

## 3. Target Detection-Based Algorithm for Rail Transit Equipment Inspection

In response to the characteristics and requirements of rail transit equipment inspection tasks, we selected the YOLO object detection algorithm to assist in the implementation of the inspection task. Specifically, we adopted the latest YOLOv8 object detection model, fine-tuned on our custom dataset, in the intelligent rail transit equipment inspection system. To further enhance the accuracy of equipment status recognition and ensure the stability of detection results, we introduced the ResNet-18 deep residual network model, also fine-tuned, for the equipment status detection sub-task.

### 3.1. Deep Learning Model Selection and Architecture

The selection of YOLOv8 and ResNet-18 was driven by a rigorous trade-off analysis between computational latency, detection accuracy, and deployment feasibility on edge devices (NVIDIA Jetson AGX Orin). While other architectures such as MobileNetV2 offer lower latency, our preliminary analysis indicated they lacked the feature extraction depth required for detecting small mechanical defects in low-light tunnel environments. Conversely, larger models like ResNet-50 or YOLOv8-L incurred inference latencies exceeding our 33 ms real-time threshold.

**Figure 3:**
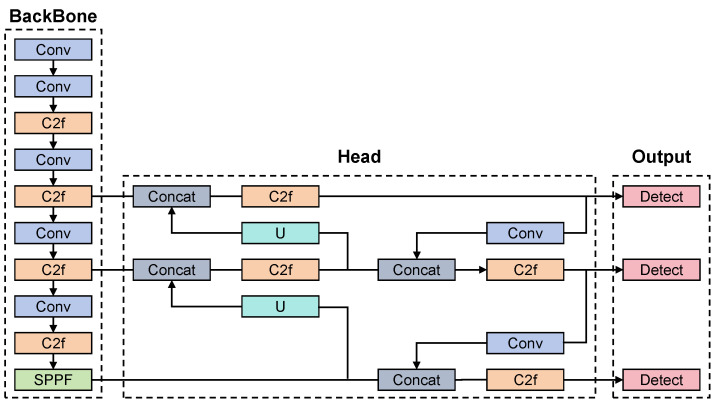
YOLOv8 Architecture Diagram [9].

YOLOv8 for Detection: We adopted the YOLOv8 object detection model (Figure 3) for the initial localization of personnel and equipment. YOLOv8 was chosen due to its anchor-free head and improved loss function, which provide superior performance in identifying small objects compared to previous iterations. We specifically fine-tuned the detection head to recognize rail-specific equipment classes.

**Figure 4:**
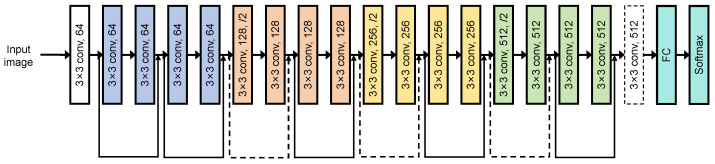
ResNet-18 structure [23].

ResNet-18 for Classification: For the subsequent status classification stage, ResNet-18 (Figure 4) was selected over shallower networks. Its residual connections prevent gradient degradation, allowing for robust feature learning without the heavy computational cost of deeper variants like ResNet-101. This method allows us to accurately determine the status (e.g., Open/Closed) of the detected equipment regions.

### 3.2. Real-Time Inspection Personnel Monitoring Algorithm Design

**Figure 5:**
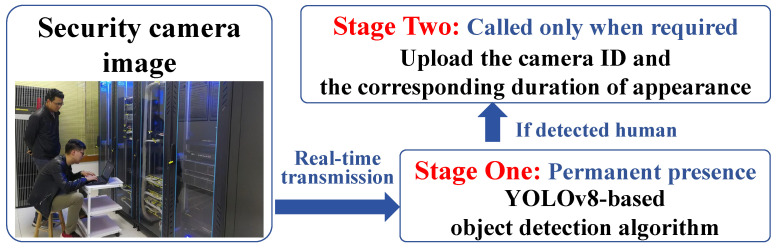
Architecture of the proposed two-stage algorithm.

The real-time inspection personnel monitoring algorithm, based on object detection, utilizes the YOLOv8 model to detect objects appearing in the surveillance camera images in real-time, especially when inspection personnel appear in the frame, enabling accurate identification and localization. The core function of this algorithm is to record the appearance time and total stay time of inspection personnel in the surveillance frames, in order to assess their work status. The overall architecture of the algorithm is shown in Figure 5, which demonstrates how the algorithm is efficiently integrated into the surveillance system, supporting the maintenance and management of rail transit equipment.

We provide the pseudocode in Algorithm 1, offering a high-level logical overview of the algorithm. By designing the object detection model, we are able to separate the tasks of detecting the presence of inspection personnel in the monitoring frames and recording their appearance time. When no inspection personnel appear in the frame, the system only runs the YOLOv8 object detection network. This increases the frame rate of the model, enhances detection capabilities, and reduces unnecessary computations. Once inspection personnel are detected in the frame, the system automatically begins recording the time they first appear in the frame and their total stay duration. This design allows the model to run efficiently when no data needs to be recorded, while accurately recording key time data when inspection personnel are detected, thus effectively evaluating the work status of the personnel.

Under the cloud-edge-end collaborative architecture based on edge intelligence, the real-time inspection personnel monitoring algorithm can be fully deployed and run on the edge server, enabling the detection of the work status and workflow of inspection personnel. In the rail transit equipment inspection process, we deploy surveillance cameras in key areas and use advanced algorithms to monitor and evaluate the work status of inspection personnel in real-time. Specifically, we first define the number of devices that each camera frame should cover and set the time range required for inspecting each device. The algorithm begins timing from the moment the inspection personnel enter the camera frame until they leave the frame, recording the actual inspection time. The algorithm then compares this actual time with the predefined time range. If the actual time is within the range, the work status of the personnel is considered normal; if it exceeds the range, whether too short or too long, the algorithm will flag it as an anomaly and alert the staff at the data processing center.

Moreover, in the rail transit equipment inspection process, we ensure that the inspection personnel follow the established inspection sequence by numbering the surveillance cameras. The personnel must appear in the camera frames in the numerical order of the cameras to complete the correct inspection process. The algorithm monitors this process by recording the order in which inspection personnel appear in each camera frame. Each time inspection personnel enter a camera frame, the camera number is added to an array. This array is then compared with a reference array that contains the camera numbers arranged in the prescribed inspection order. If the recorded camera numbers match those in the reference array, the inspection process is considered normal. Any inconsistency will trigger a detection of missed or incorrect inspections and automatically alert the staff at the data processing center. This allows real-time monitoring of the personnel’s work and ensures that the rail transit equipment inspection process is conducted properly.
**Algorithm 1:** Personnel Presence and Operation Monitoring Algorithm
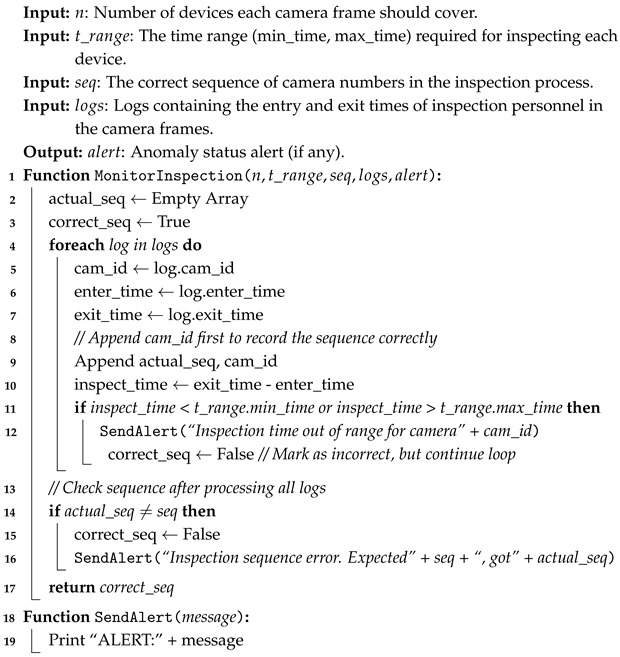


### 3.3. Rail Transit Equipment Status Detection Algorithm Design

**Figure 6:**
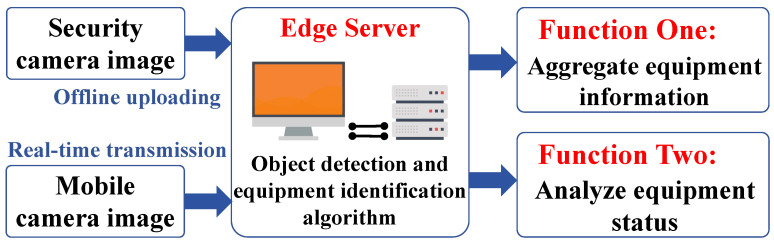
Architecture of the proposed target identification algorithm.

The rail transit equipment status detection algorithm, based on object detection, utilizes the YOLOv8 model to detect and localize rail transit equipment in video recorded by mobile cameras and surveillance frames. Furthermore, we employ a regression network based on ResNet-18 to detect the equipment’s status, such as whether the lid of tunnel trackside equipment is open or whether the door of the equipment room is open. The overall architecture of this algorithm is shown in Figure 6.

We provide the pseudocode in Algorithm 2, offering a high-level logical overview of the algorithm.
**Algorithm 2:** Subway Communication Equipment Identification and Status Detection Algorithm
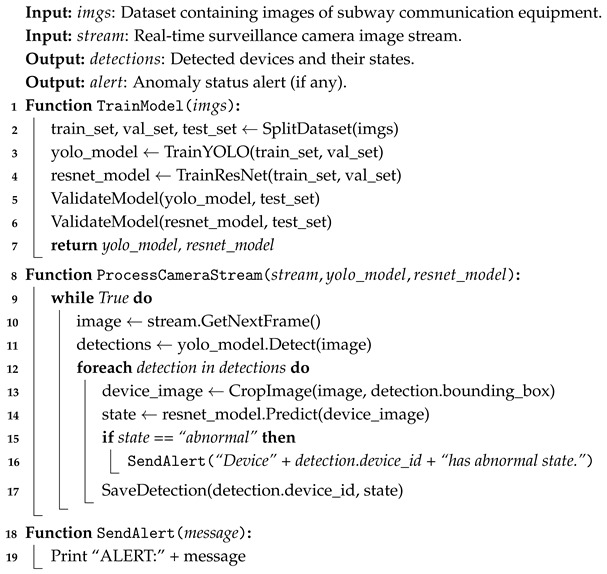


Under the cloud-edge-end collaborative architecture based on edge intelligence, we can fully deploy and run the rail transit equipment status detection algorithm on the edge server, enabling the detection and status evaluation of subway communication equipment. For the detection and recognition function of subway communication equipment, we precisely annotate each type of equipment that needs to be detected, compile and use a dataset for model training and validation, and finally deploy the trained model on the edge server for quick and accurate detection of subway communication equipment. In practice, we use an advanced algorithm to determine the open or closed state of equipment cabinet doors. We chose ResNet-18 as the base model for state detection. ResNet-18 addresses the accuracy saturation and degradation issues that deep convolutional neural networks (CNNs) may face during training through residual learning [24,25]. The core advantage of ResNet-18 lies in its residual units, which allow the network to learn residual functions between inputs and outputs, making training effective even for very deep networks. After preprocessing, RGB images (cropped by YOLOv8) are fed into the ResNet-18 network, processed through a series of convolutional layers and residual blocks. The output features are passed to a 512-unit fully connected layer, which is then connected to a final 2-unit dense layer with softmax activation to predict the “normal” or “abnormal” state of the equipment door. By combining ResNet-18 with YOLOv8, we achieve a robust, two-stage pipeline for precise detection of rail transit equipment status. YOLOv8 first handles the localization of equipment in the image, and ResNet-18 then performs a fine-grained analysis of the cropped region to determine its open or closed status. This combination leverages the strengths of both models, providing a comprehensive solution for state monitoring.

## 4. Experiment Results and Discussion

In this section, we evaluate the proposed system architecture and inspection algorithms through a combination of field deployment and simulation. First, we deployed the system at Wuzixueyuan Road Station and Jishuitan Operation Center of Beijing Subway Line 6. Second, we conducted discrete-event simulations to stress-test the offloading scenarios (local, edge, and cloud) under varying loads. Finally, we trained the recognition models using the dataset described in Table 1 and validated the intelligent inspection algorithm in real-world conditions. All reported model performance metrics (e.g., mAP, accuracy) represent the mean of 5 independent training runs to ensure statistical reliability.

### 4.1. Experimental Setup and System Architecture Test

To validate the proposed architecture, we established a comparative experimental environment. The edge server is equipped with an NVIDIA Jetson AGX Orin 64 GB module (12-core Arm Cortex-A78AE CPU, 2048-core NVIDIA Ampere GPU), provided by NVIDIA Corporation, Santa Clara, CA, USA, representing high-performance edge computing. For the cloud baseline, we utilized an AWS EC2 p3.2xlarge instance equipped with an NVIDIA V100 GPU, provided by Amazon Web Services (AWS), Seattle, WA, USA, to simulate centralized processing.

**Figure 7:**
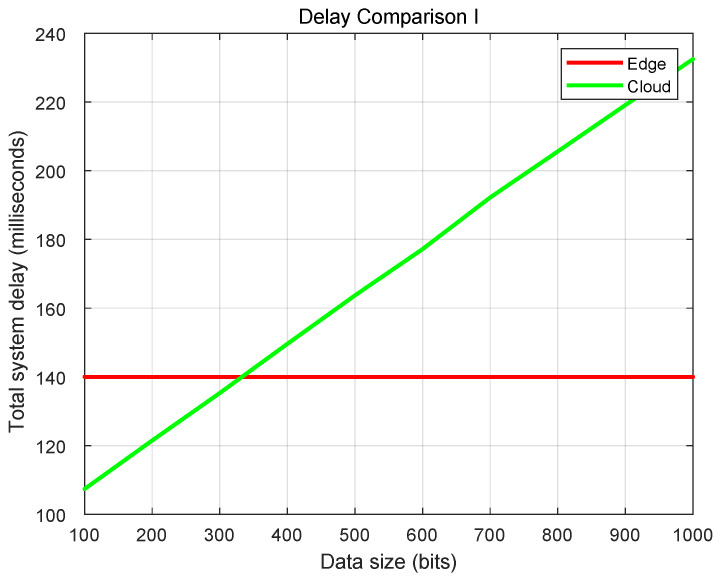
Latency test results with varying task data sizes.

**Figure 8:**
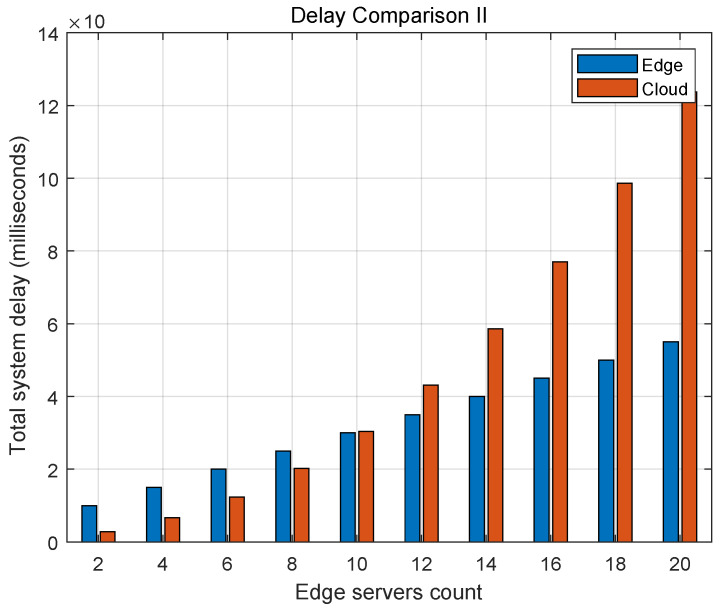
Latency test results with varying number of edge servers.

It is important to note that the latency results presented in Figure 7 and Figure 8 were obtained through discrete-event simulation to assess the scalability of the architecture under loads that exceed current physical deployment capabilities. The x-axis unit refers to the normalized packet payload size in the simulation model. This theoretical modeling explains the linear characteristics observed in the delay curves and serves to benchmark the architectural limits.

Figure 7 shows the system latency variation with task data size, assessing the impact of task data size on the offloading strategy. When all tasks are offloaded to the edge server, the system’s overall latency remains stable, as latency is only related to the computational power of the edge server. In contrast, in the cloud offloading scenario, as the data size increases, communication delays increase significantly, leading to higher overall computation delays. Especially when the computation task exceeds 320 bits, the latency of edge computing outperforms cloud computing, demonstrating the advantage of edge computing.

Figure 8 illustrates the system latency variation with the number of edge servers. With more than 10 edge servers, the latency of edge computing remains consistently lower than that of cloud computing. This confirms that edge offloading significantly reduces the total system latency, improving real-time performance while lowering energy consumption.

### 4.2. Field Deployment and Testing of the Algorithm

To verify the feasibility of our algorithm, we deployed the system on Beijing Subway Line 6. Surveillance cameras were deployed in the communication equipment room and held by interval inspection personnel. All models were fine-tuned using the hyperparameters specified in Table 2.

**Figure 9:**
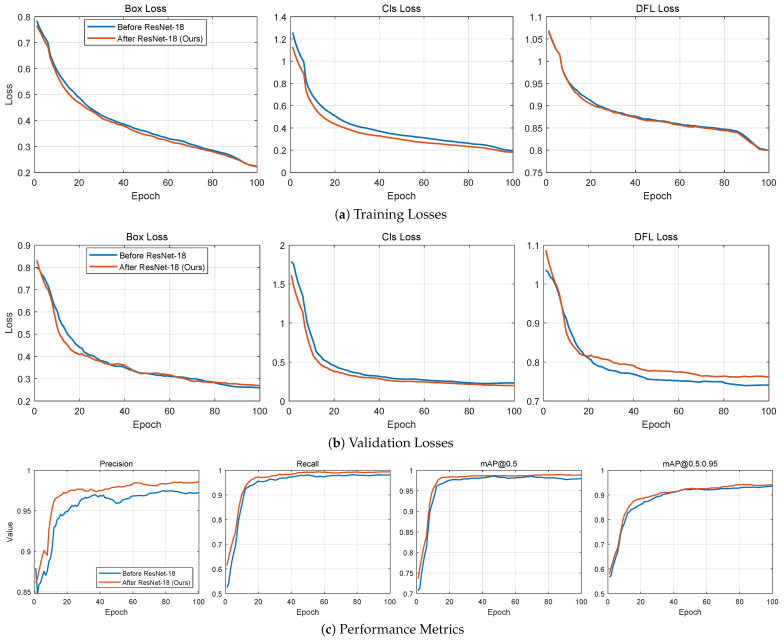
Recognition Model Training Results.

Figure 9a,b show the training results of the model with and without ResNet-18, respectively. The results indicate that after incorporating ResNet-18, the model performs more stably and excellently across various training and validation metrics: training loss changes smoothly, validation loss fluctuates minimally, showing strong generalization ability; accuracy and recall are well-balanced, and mAP50 and mAP50-95 consistently improve across multiple training epochs, indicating strong and stable detection capabilities; learning rate adjustment is stable, aiding in model optimization. Although the control group showed a reduction in training loss, the loss and mAP in the validation set exhibited significant fluctuations, indicating overfitting.

During the field deployment, we specifically analyzed system behavior under non-ideal conditions. The system demonstrated occasional performance degradation in scenarios involving extreme motion blur caused by rapid movement of handheld devices, which temporarily prevented the YOLOv8 model from locking onto equipment boundaries. Additionally, complex lighting variances (e.g., sudden train headlights) occasionally triggered false positives in the “Abnormal” status classifier. However, due to the high frame rate (25 FPS) and temporal redundancy logic, the system typically corrected these detections within 3–5 frames (approx. 150 ms), maintaining high overall availability.

The quantitative performance of the personnel monitoring logic (Algorithm 1) was validated on a test set of 100 simulated inspection videos, achieving 98% accuracy (Table 3).

To evaluate the overall performance of the proposed equipment status detection algorithm (Algorithm 2), we combined the confusion matrix analysis with quantitative metrics. Table 4 presents the detailed classification results of the ResNet-18 model and the corresponding performance metrics.

As shown in Table 4, the model demonstrates high reliability. Out of 160 actual ‘Close’ instances, 159 were correctly predicted. For the critical ‘Open’ (Abnormal) class, the system achieved a recall of 99.3%, missing only 1 instance. The slight confusion with the ‘Background’ class (3 instances) indicates minor challenges in complex lighting, which are mitigated by temporal redundancy in the video stream.

### 4.3. Network Bandwidth and Storage Efficiency Analysis

To address concerns regarding the practical utility of EI in bandwidth-constrained rail environments, we conducted a rigorous comparative analysis of uplink data traffic. Unlike traditional studies that compare EI against unoptimized legacy systems, we benchmark our system against a highly optimized Cloud Baseline utilizing H.265+ Smart Coding to ensure a fair evaluation of architectural efficiency rather than compression differences. We assume a typical deployment of 5 cameras per station node.

- Cloud Baseline (Optimized): This scenario assumes 5 cameras (1920 × 1080, 25 FPS) streaming continuously (24 h) to meet security compliance. By enabling H.265+ Smart Coding, the bitrate for largely static rail scenes is reduced to an average of 0.74 Mbps per camera.
- EI System (Enhanced): The edge server processes video locally. To ensure auditability for safety-critical anomalies, the system uploads data only upon event detection. Each upload consists of one high-resolution image (300 KB) and a 10-s video clip (H.265 encoded, ≈2.5 MB) as evidence. We assume a high-traffic scenario with approximately 270 trigger events per day (including routine hourly health checks and personnel/anomaly detections).

As shown in Table 5, even when compared against an optimized H.265+ baseline, the proposed EI system reduces daily data traffic from 40 GB to 0.76 GB. This 98.1% reduction is critical for rail transit tunnels where 5G/4G signal strength can be intermittent. Furthermore, the inclusion of 10-s video evidence in the EI payload addresses the practical need for human verification without incurring the massive overhead of continuous streaming.

**Figure 10:**
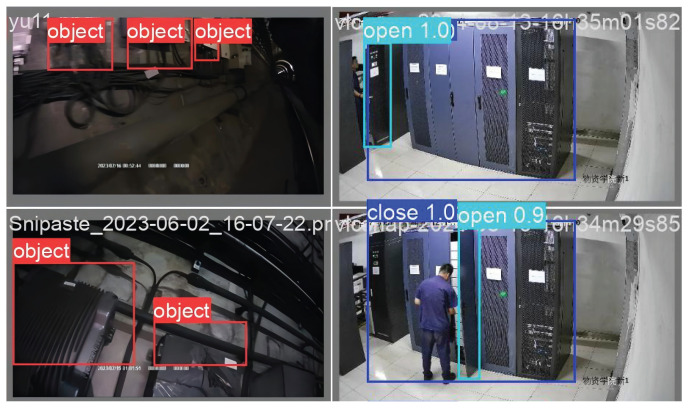
Intelligent Inspection System Operation Results. (The Chinese text in the figure represents the watermark of the camera used for dataset collection).

During the trial operation phase, we participated in the daily work of inspection personnel and used the intelligent inspection algorithm to monitor both the work status of the personnel and the operational status of the rail transit equipment. As shown in Figure 10, the intelligent inspection system accurately and quickly recognizes the equipment and its status. In the actual operation, the system was benchmarked against manual review of the same video feeds, achieving a 98% accuracy in detecting abnormal states (such as open cabinets) with an average end-to-end detection-to-alert latency of 28.5 ms on the edge server. This performance validates its effectiveness for real-world deployment.

## 5. Conclusions

This study presented a rail transit equipment inspection system based on Edge Intelligence (EI) and 5G technology, designed to address the high cost, error rate, and inefficiency of traditional manual inspection methods. By adopting a cloud–edge–end collaborative architecture compliant with the ETSI MEC framework, the system effectively enhanced detection speed and accuracy while optimizing data processing and transmission efficiency. Experimental results demonstrated that the EI-based architecture reduced system latency by 45% compared with a cloud-centric baseline (28.5 ms vs. 52.1 ms). Field tests conducted on Beijing Metro Line 6 validated the proposed two-stage algorithm. The fine-tuned YOLOv8n model achieved 92.7% mAP@0.5 for equipment detection, while the ResNet-18 classifier attained 95.8% accuracy for status classification. Moreover, the personnel monitoring module demonstrated 98% accuracy in detecting procedural anomalies.

Beyond latency reduction, the EI architecture further strengthened system performance in three critical dimensions. First, accuracy was improved by processing lossless raw video streams at the edge, eliminating detection errors introduced by cloud-side compression artifacts. Second, security was enhanced through the deployment of a 5G private network that isolated sensitive operational data from the public internet. Third, the system was designed to improve robustness by leveraging distributed edge nodes, which conceptually avoids single points of failure and maintains operational continuity even under partial node degradation.

Critically, the revised bandwidth analysis revealed that the EI system reduced daily uplink data consumption by roughly 98.1% (from 40.0 GB to 0.76 GB) compared to a cloud-based streaming baseline. By selectively transmitting short video evidence only when anomalies were detected, the system ensured both bandwidth efficiency and sufficient evidential fidelity for human verification. Overall, the proposed system demonstrated measurable improvements in inspection efficiency, accuracy, security, and architectural resilience, while reducing labor costs and communication overhead.

Despite these promising results, the current system remained limited to binary (normal/abnormal) anomaly classification and data collected from a single metro line. Furthermore, the current robustness analysis is primarily based on architectural design rather than stress testing. Future work would focus on developing multi-level anomaly severity classification—such as distinguishing critical signal failures from minor cabinet issues—and expanding the dataset to cover a wider range of equipment types and environmental conditions. In addition, we plan to conduct dedicated fault-injection experiments and reliability modeling to quantitatively assess the system’s performance under edge-node failure and network partition scenarios, thereby validating its practical robustness in extreme conditions.

## Figures and Tables

**Table 1:** The Rail Transit Equipment Inspection Dataset.

Item	Description
Collection Period	January–June 2024
Location	Beijing Metro Line 6 (Wuzixueyuan Rd, Jishuitan)
Equipment	Hikvision DS-MH2311-G (mobile), DS-2CD3T86FWD (fixed)
Resolution	1920 × 1080 pixels at 25 FPS
Scene Types	Track sections and equipment rooms (varying light)
Total Images	10,240 images
Annotation Classes	1. Personnel (2048 images, 20%)
	2. Equipment (8192 images, 80%)
Equipment Sub-classes	Signal Cabinet (Normal/Abnormal), Trackside Box (Open/Closed)
Normal/Abnormal Def.	Normal: Cabinet door fully closed and latched.
	Abnormal: Cabinet door ajar, open, or visible damage.
Dataset Split	Training: 70% (7168), Validation: 15% (1536), Testing: 15% (1536)
Data Augmentation	Random Flip (horizontal), Rotation (±10 deg), Mosaic
Labeling Protocol	CVAT; Inter-annotator reliability: 0.92 IoU

**Table 2:** Model Training Hyperparameters.

Parameter	YOLOv8n	ResNet-18
Pre-trained Weights	COCO	ImageNet
Optimizer	Adam	Adam
Learning Rate	1 × 10^−4^	1 × 10^−4^
Batch Size	32	32
Epochs	50	20
Image Size	640 × 640	224 × 224

**Table 3:** Quantitative Performance of Algorithm 1 (Personnel Monitoring).

Metric	Value
Test Scenarios	100 sequences
Correct Detections	98
False Positives (False Alarms)	1
False Negatives (Missed Alarms)	1
Anomaly Detection Accuracy	98.0%

**Table 4:** Comprehensive Performance Evaluation of Algorithm 2 (Equipment Status Classification).

(a) Confusion Matrix (ResNet-18 Status Classification)					
		**Predicted Class**			
		Close	Open	Background	Total
True Class	Close	552	10	0	562
	Open	20	200	0	220
	Background	0	3	0	3
(b) Quantitative Metrics (Mean ± SD)					
**Metric**			**Value**		
Detection mAP@0.5 (YOLOv8n)			92.7% ± 0.4%		
Precision			0.94 ± 0.02		
Recall			0.91 ± 0.03		
F1-Score			0.92 ± 0.02		
Classification Accuracy (ResNet-18)			95.8% ± 0.3%		

**Table 5:** Daily Bandwidth Consumption Comparison (5 Cameras).

Parameter	Cloud Baseline (Optimized)	EI System (Enhanced)
Video Encoding	H.265+ Smart Codec	H.265 (Clip)
Transmission Mode	Continuous (24 h)	Event-Triggered
Per Event Payload	N/A	Image + 10 s Video (≈2.8 MB)
Daily Data Volume	≈40.0 GB	≈0.76 GB
**Bandwidth Saving**	**98.1% Reduction**	

## Data Availability

Data is unavailable due to privacy restrictions.
